# A New Framework to Interpret Individual Inter-Hemispheric Compensatory Communication after Stroke

**DOI:** 10.3390/jpm12010059

**Published:** 2022-01-06

**Authors:** Arianna Brancaccio, Davide Tabarelli, Paolo Belardinelli

**Affiliations:** Center for Mind/Brain Sciences—CIMeC, University of Trento, I-38123 Trento, Italy; arianna.brancaccio@unitn.it (A.B.); davide.tabarelli@unitn.it (D.T.)

**Keywords:** stroke, inter-hemispheric communication, individualized stimulation rehabilitation protocols, callosal integrity, diaschisis, frontal control network, brain stimulation

## Abstract

Stroke constitutes the main cause of adult disability worldwide. Even after application of standard rehabilitation protocols, the majority of patients still show relevant motor impairment. Outcomes of standard rehabilitation protocols have led to mixed results, suggesting that relevant factors for brain re-organization after stroke have not been considered in explanatory models. Therefore, finding a comprehensive model to optimally define patient-dependent rehabilitation protocols represents a crucial topic in clinical neuroscience. In this context, we first report on the rehabilitation models conceived thus far in the attempt of predicting stroke rehabilitation outcomes. Then, we propose a new framework to interpret results in stroke literature in the light of the latest evidence regarding: (1) the role of the callosum in inter-hemispheric communication, (2) the role of prefrontal cortices in exerting a control function, and (3) diaschisis mechanisms. These new pieces of evidence on the role of callosum can help to understand which compensatory mechanism may take place following a stroke. Moreover, depending on the individual impairment, the prefrontal control network will play different roles according to the need of high-level motor control. We believe that our new model, which includes crucial overlooked factors, will enable clinicians to better define individualized motor rehabilitation protocols.

## 1. Introduction

Stroke incidence is increasing in developing countries and young populations [1,2]. Despite its widespread occurrences, stroke still represents the principal condition leading to adult disability and motor impairments, even after the application of rehabilitation protocols [3]. Two main types of strokes are usually reported: ischemic and hemorrhagic stroke, with ischemic stroke constituting the 80% of the cases [4]. In most cases, strokes are associated with damage at the level of the middle cerebral artery [5]. This structure consists of branches of vessels that provide blood supply to both cortical and subcortical structures. For this reason, strokes are further distinguished between cortical and subcortical ones, according to the mostly damaged areas. In general, a sudden arrest in blood supply to neuronal cells which die from oxygen deprivation is at the basis of the stroke syndrome [4]. In the clinical practice, different brain stimulation protocols, together with standard physical rehabilitation practices, are delivered to the patient in order to increase the level of recovery after the stroke. However, in a remarkable number of cases, the patients still show impairment, even following the rehabilitation treatment. In this context, results reported in literature are still inconsistent since key factors have thus far not been considered in modeling post-stroke brain re-organization. This review proposes a new theoretical framework to better account for results in stroke literature, with a particular focus on the effectiveness of the different stimulation motor rehabilitation protocols proposed thus far.

Non-invasive brain stimulation (NIBS) protocols have been mainly inspired by currently available models of inter-hemispheric communication in strokes. The three main models are the inter-hemispheric competition model, the vicariation model, and the bimodal balance–recovery model. We think that, at present, these models need a critical revision. From a purely anatomical perspective, none of these models takes into account diaschisis processes that occur in response to the lesion. In particular, we hereby refer to transcallosal and contralesional diaschisis. In this context, diaschisis refers to structural changes in regions that have not been directly affected by the lesion but degenerate because they are indirectly connected to the lesioned site [6]. Therefore, a revision explaining how both hemispheres cope with the lesion is required, since the evidence of such significant anatomical changes as a response to the lesion is recent and was not available at the time when these models were proposed. On the other hand, functional changes in brain network re-organization need to be included as well. In particular, the role of prefrontal cortices should be considered, given that most stroke patients show differences in terms of functional connectivity, especially in the fronto-parietal network (e.g., [7,8]).

The role of structural reserve was first proposed by Di Pino and colleagues (2014) who tried to encompass the mixed results obtained by stimulation protocols inspired by the previous models in a more general framework [9]. Here, we stress the importance of the concept of structural reserve and propose to look at stroke severity on a continuous range, from the least to the most affected patients, with the mildly affected ones showing types of functional reorganization more similar to the minimally or the severely affected ones, depending on the kind of offence suffered. We believe that the allocation of a patient on a continuum of severity depends on the structural damage as well as on diaschisis processes of tissues connected to the lesion. Critically, depending on the spared tissue, functional reorganization of cortical networks will either afford to resolve compensation within the ipsilesional hemisphere or, in more severe cases, will have to rely on the contralesional hemisphere. Crucially, we propose that the structural reserve in the corpus callosum (CC) has to be considered to understand which type of reorganization the patient will possibly undertake. Furthermore, we suggest that functional brain network reorganization does not exclusively encompass sensorimotor areas, but the prefrontal network may also access a higher-level control over motor planning and execution. Importantly, while collecting all the pieces of evidence necessary to develop a more complete framework of stroke recovery, we also stress the epistemological mistakes that have been committed in interpreting stroke results. In fact, classic literature of stroke recovery has associated bad motor recovery to specific reorganization patterns that were reported in severely affected patients and thus thought to be maladaptive. Differently, we propose to look at these cases as the expression of the best possible reorganization the brain can undertake in order to gain some degree of recovery.

This review is focused on motor impairment and motor rehabilitation in stroke patients. The choice to focus only on the motor aspects of post-stroke deficits depends on two reasons. First, the most common impairment after stroke consists in motor deficits of the affected side of the body, with more than 80% of patients showing motor impairment in the acute stage and more than 40% in the chronic stage (e.g., [10]). The second reason as to why we chose to focus on motor recovery, neglecting the cognitive aspects of stroke impairment, depends on the fact that the literature abounds in works investigating the relationship between motor scores on the assessment scales and connectivity patterns. However, at the same time, few works are available on the relationship between scores on cognitive assessment scales and connectivity patterns in stroke patients. For this reason, a comprehensive systematization on the relationship between cognitive impairment and connectivity patterns would have been inevitably incomplete.

This review is organized as follows. In the second section, we discuss the main stroke recovery models that have inspired recent rehabilitation protocols. In the third section, we report a new explanatory model of inter-hemispheric interplay according to which the two hemispheres do not compete, but rather constantly integrate information via both facilitation and inhibition. In the fourth section, we critically analyze the current status of literature, showing the relevance of late discoveries regarding the role of corpus callosum and prefrontal cortices in sustaining stroke recovery. In the fifth section, we describe our new framework that delineates the different possibilities in stroke reorganization patterns. Finally, in the last part of this review, we outline how individual, patient-tailored rehabilitation protocols can be developed on the basis of our new framework.

## 2. Stroke Recovery Models and Efficiency of Non-Invasive Rehabilitation Protocols

Transcranial magnetic stimulation (TMS) and transcranial direct current stimulation (tDCS) techniques have been used in the past years to improve motor recovery in stroke patients, often jointly with standard physiotherapy practice. However, while at the single study level the administration of TMS and tDCS treatments appear beneficial to patients in motor and/or cognitive domain, meta-analysis results have shown that these effects are rather limited or, in some cases, even non-existent (e.g., [11,12,13,14,15]). It should be noticed that these stimulation protocols have been developed on the basis of the conviction that either the interhemispheric competition or the vicariation model might be the one that best predicts recovery [9].

The interhemispheric competition model was first introduced by Cook in 1984 [16]. It assumes that the two hemispheres are in a continuous rivalry condition, by which they constantly inhibit each other via transcallosal connections to allow functional lateralization and avoid maladaptive interhemispheric crosstalk [17]. This hypothesis of interhemispheric competitive interplay has led to the view that, when a stroke lesion damages one hemisphere, the unaffected one takes the lead by exercising excessive inhibition over the damaged one, while receiving in turn insufficient inhibition from the damaged one; this should lead to an excessive depression of the spared activity in the affected region. Therefore, the affected hemisphere suffers from both damage and excessive inhibition exerted by the non-lesioned hemisphere. TMS and tDCS rehabilitative protocols inspired by the interhemispheric competition model hence focused on limiting the inhibition exerted by the unaffected hemisphere and/or enhancing excitability of the affected one (e.g., [18,19]). Contralesional inhibition is supposed to decrease the inhibitory action of the spared hemisphere on one hand, while ipsilesional facilitation is thought to facilitate the inhibitory influence from the affected one [20]. Evidence in support of this model stems from studies showing that (1) stroke patients attempting to produce a movement with the paretic hand fail because the unaffected hemisphere keeps inhibiting the affected one and does not switch to the facilitation mode at the time of movement onset [21]; (2) anesthesia reducing somatosensory input from one hand leads to an improvement in tactile spatial acuity for the contralateral hand [22]; and (3) inhibitory repetitive TMS (rTMS) protocols applied on the contralesional motor cortex in some cases lead to an improvement in the paretic hand motor control [23]. However, the evidence regarding the effectiveness of rTMS and tDCS rehabilitative protocols based on the competition model are inconclusive when results are evaluated at meta-analysis level. A review by Klomjai and colleagues (2015) has collected results of four main meta-analyses on rTMS and tDCS protocols for stroke rehabilitation. Of these four meta-analyses, three focused on the use of rTMS: while one of them did not find any evidence of a successful application of rTMS protocols for stroke rehabilitation, two meta-analyses suggested a mild positive effect for subcortical strokes when inhibitive, low-frequency (1 Hz) rTMS was applied over the contralesional hemisphere [24,25,26,27]. Another recent and comprehensive review [28], including 112 TMS studies, evidenced the need of revising protocols inspired by the competition model. Indeed, the authors did not find (1) any evidence for a hyper-excitability of the unaffected hemisphere in stroke patients or (2) any proof in favor of an inter-hemispheric, maladaptive imbalance. If the inter-hemispheric competition model were correct, the unaffected hemisphere should show hyper-excitability, given the impossibility for the affected hemisphere to participate in the process of mutual inhibition.

The vicariation model represents the alternative to the competition model and has been proposed in the attempt to explain how the brain might be coping with a stroke lesion (e.g., [29]). This model suggests that the over-activation of the contralesional hemisphere may be not maladaptive, but rather a vicarious mechanism through which the non-lesioned hemisphere compensates for the affected one’s functional and structural damage. In this regard, a study by Johansen-Berg and colleagues (2002) showed that when TMS is used to disrupt the activity in the premotor cortex of the contralsional hemisphere, ipsilateral reaction time was slowed down by 12% in stroke patients but not in the control group, indicating a role of the contralesional hemisphere in sustaining motor production of the ipsilateral limb. This effect was greatest in the most impaired patients, suggesting that the larger the damage, the more the contralesional hemisphere is necessary for functional compensation. Further evidence in support of the vicariation model reveals that well-recovered chronic stroke patients show contralesional recruitment during motor implementation [30,31]. Furthermore, it has been shown that suppressive cathodal tDCS over the contralesional hemisphere in mildly impaired patients led to an improvement in the ipsilateral motor functions, while the same stimulation applied on severe patients worsened the motor outcome [32]. Taken together, these results were interpreted as supportive evidence that the vicariation process might intervene when the affected hemisphere is strongly damaged. Indeed, in case of less severe damage, the plastic reorganization supporting recovery is thought to be manageable within the lesioned hemisphere itself, with no necessity of a more radical reorganization involving the unaffected one [33]. However, at the meta-analysis level, slightly positive experimental evidence of stroke patients’ recovery was found for protocols that facilitate either the affected hemisphere in mildly affected patients or the unaffected one in severely affected patients [28]. Moreover, the supportive role of the unaffected hemisphere has been reported for both moderate and severely affected chronic stroke patients [34]. Furthermore, the role of the unaffected hemisphere in sustaining recovery independently on the level of damage has been considered not only in motor functions, but also in recovery from oropharyngeal dysphagia. In fact, Park and colleagues found that high-frequency (5 Hz) rTMS over the contralesional hemisphere leads to reduced dysphagia in stroke patients independently on the level of damage [35].

Prior to the evidence from meta-analyses on the ineffectiveness of protocols inspired by the competition model [28], an attempt to explain and integrate the mixed results in favor of one or the other model was made with the *bimodal balance-recovery model* by Di Pino and colleagues (2014). This framework advocates both competition and vicariation models as explanatory depending on the level of structural damage in the individual patient, where structural integrity is described as “*the remaining function of the motor areas and corticospinal tract in the affected hemisphere*”. The authors suggest that interhemispheric competition predicts recovery more accurately in cases where the structural reserve is high. In a nutshell, less interhemispheric imbalance predicts better recovery for patients with limited damage. Differently, when the damage in the affected hemisphere is too spread out to attempt a local effective recovery, the over-recruitment of the unaffected hemisphere reflects its vicarious contribution. Even though this model was never directly tested, some works have shown that patients can be distinguished into sub-groups according to the level of damage. Moreover, this distinction predicts better recovery either after inhibitory tDCS over the affected hemisphere or excitatory stimulation of the unaffected one, suggesting that the mechanisms at the basis of an improvement after the lesion might change according to the amount of damage [9,36].

However, the effort of Di Pino and colleagues to encompass different recovery models in one operative framework appears faulty in light of recent meta-analyses reporting no statistical evidence in support of the competition model [28]. Moreover, Di Pino and colleagues did not consider the high-level control from prefrontal cortices, the extent of which can help to distinguish between different types of patients on the basis of their respective reorganization patterns. The interhemispheric competition model constitutes an attempt at understanding the general mechanism of interaction between the hemispheres. The vicariation model, on the other hand, cannot be considered as a general model of brain functioning but rather as an endeavor to explain plastic changes unburdening stroke damage. The attempt of simply comprising both competition and vicariation model in the biphasic model reflects a knowledge gap and can be considered as a by-product of the fact that the general mechanism of interhemispheric communication is thus far still unclear. In particular, the question as to whether the two hemispheres are in an inhibitory or facilitatory relationship and to what extent one mechanism takes over the other is still an open matter.

For the sake of completeness, the authors hereby report two further attempts at improving stroke patients’ stratification. Differently from the interhemispheric competition, vicariation, and biphasic models, these pipelines represent empirical models not focused on explanation of post-stroke interhemispheric interplay (i.e., inhibition or vicariation), but rather on finding a sensitive way to organize information from the individual patient in order to predict recovery and stimulation protocol outcomes. These two algorithms are the Predicting REcovery Potential (PREP) and the Structural reserve, Task Attributes, Connectivity (STAC). The PREP was introduced prior to the biphasic model, while STAC is the most recent one. For this reason, the main difference between the two models lies in the fact that STAC explicitly integrates the idea by Di Pino and colleagues, according to which the level of damage constitutes a weighting factor to choose the best stimulation protocol, and not only to assess the level of recovery [37]. In this sense, the bimodal balance-recovery model can be considered as a watershed in stroke literature.

The PREP algorithm [38] stratifies patients in four categories: those who will go towards a “complete”, “notable”, “limited”, or “none” recovery. The evaluation of a patient’s potential recovery involves different steps, from the motor evaluation to the use of diffusion-weighted MRI to assess structural reserve in patients who show poor scores and no MEPs in TMS trials. PREP showed a predictive power of 88% and hence was proposed as an efficient method for predicting recovery. However, as previously mentioned, this algorithm is not informative about the type of NIBS protocol that would best suit a specific patient.

On the other hand, STAC takes its acronym from the weighting factors that the authors propose as relevant when choosing for the best stimulation rehabilitative protocol. According to the authors, the structural reserve in the cortico-spinal tract (CST) needs to be used as a weighting factor to decide the best rehabilitative protocol. The innovation of their proposal lies in the criterion for choosing the best stimulation site: structural integrity needs to be considered jointly with two further factors, namely, (1) the task attributes and (2) the structural/functional connectivity of the candidate sites. As for the task attributes, the authors stress that different rehabilitative tasks involve different neural networks. Specifically, each task recruits different limbs’ segments, and therefore the structural and functional features of, e.g., proximal and distal muscles can differ. Hence, different stimulation sites should be chosen according to the task. To intervene on structural and functional intra/interhemispheric connections (last considered factor), a precise stimulation over circumscribed cortical areas is recommended. To assess connectivity patterns of a region, structural connectivity analyses (e.g., DW-MRI) and functional techniques (e.g., non-invasive priming) are recommended.

Differently from previous pipelines, STAC does not rely on a general model explaining plastic changes in stroke patients but is rather a data-driven approach focused on finding a protocol that depends on patient’s status, lesion site, and task specific factors. Furthermore, STAC considers interhemispheric structural connectivity only with the aim to better target a specific stimulation site. This approach is expanded in this work, proposing a new general model that could also be efficiently employed in a clinical environment. Individual interhemispheric structural connections should be used not only to understand whether a specific area needs to be stimulated, but most importantly to assess the spared callosal pathways which can be exploited to support interhemispheric integration.

## 3. Inhibition and Facilitation Are Used to Integrate Information between Hemispheres

As reported in the previous section, the model by Di Pino and colleaguesis questionable in the light of relevant findings of the last few years [9]. First, it does not consider diaschisis processes, nor the role of the callosum into sustaining interhemispheric communication in strokes. Second, the bimodal balance–recovery model does not include a potential role of the prefrontal cortices into sustaining motor implementation with high-level control. Finally, in their model, the authors consider the competition model as correct when the structural reserve of the individual patient is sufficient for the unaffected hemisphere to not serve a vicarious role. In this regard, a recent hypothesis on callosal function has been recently proposed by Carson [39]. Carson’s hypothesis does not directly address brain reorganization after a stroke but helps to clarify the role of contralesional hemisphere in recovery. Carson supports the notion that the two hemispheres do not use either excitation or inhibition in inter-hemispheric transmission but rather both mechanisms are employed to continuously sculpt each hemisphere’s contribution to a specific behavior. In Carson’s framework, this should occur via “*crossed surround inhibition*”, which implies both excitatory and inhibitory processes. This hypothesis would explain results from works showing that stroke patients not only suffer from a motor impairment in the limb contralateral to the side of the lesion, but also the “unaffected” hand shows an impairment compared to healthy controls [40,41,42]. Furthermore, it would explain results from studies showing a negative relationship between integrity of the callosum and impairment levels in stroke patients [43,44,45,46]. This is because a greater structural integrity of CC would allow for more interhemispheric integration of information. If the inter-hemispheric competition model were correct, it should be found that less integrity in the callosum should lead to less over-inhibitory power by the unaffected hemisphere, which in turn should support a better motor status in the paretic side. However, experimental evidence shows the opposite: the higher the structural integrity of the CC, the better the motor recovery, suggesting a cooperative relationship (e.g., [43]). Moreover, Carson suggests that it is not correct to generally assume the two hemispheres in mutual competition from the outcome of TMS-derived measures such as interhemispheric inhibition and ipsilateral silent period. In fact, TMS stimulates the cortical sites at intensities largely above the natural physiological levels, thus potentially masking other key physiological processes [39]. Carson’s framework also accounts for better outcomes, which have been reported after facilitatory stimulation over the contralesional side [47,48,49]. In this regard, Wang and colleagues (2020) found motor improvement after facilitatory high frequency (10 Hz) rTMS over the contralesional hemisphere. At the same time, Ameli and colleagues (2009) found a strong positive effect of facilitatory rTMS (10 Hz) over the lesioned hemisphere. Finally, it should be noticed that induction of long-term depression-like plasticity by inhibitory stimulation (1 Hz rTMS) can be reversed to long-term potentiation depending on the excitability state of the cortex at the moment of stimulation [50]. This suggests that inhibitory stimulation can lead to functional integration depending on the level of depolarization of the neuronal population being stimulated. Carson’s hypothesis reconsiders inhibitory processes in a novel view: inhibition does not mean competition, but it can rather be leveraged cooperatively with excitation for sculpting each hemisphere’s contribution to a function. This conception of inter-hemispheric communication would also explain why the unaffected hemisphere in both mildly and severely stroke patients has been found crucial for rehabilitation [34]. Finally, the competition framework does not account for all the crossed-facilitation evidence in which the activation of one motor cortex leads to facilitation in the contralateral one (e.g., [51]). All these pieces of evidence, together with meta-analysis results regarding the incorrectness of the competition model’s assumptions, suggest that a new model where the two hemispheres are not considered as purely competing should be formulated.

## 4. Critical Review of Current Models and the Need of a More Comprehensive Framework

Our new framework was conceived in light of the latest medical evidence regarding (1) the role of the CC in sustaining inter-hemispheric functional transfer in stroke patients, considering diaschisis processes, and (2) the effects of tissue disruption leading to functional network reorganization, with a particular focus on the compensatory role of the prefrontal cortices in high-level control.

Regarding the first point, we propose that the structural reserve of CC represents an essential factor for assessing recovery in patients. Current stroke literature has mainly focused on white matter tracts different from CC, such as the internal capsule, because these were thought to be more directly affected by a stroke [52]. Nevertheless, in recent years, the damage in CC was found to lead towards altered interhemispheric communication, providing a disturbance to the plastic changes that both hemispheres undergo to endure the damage [53]. Three other pieces of evidence should be underlined: (1) a stroke lesion not only leads to neuronal death in the affected tissue, but the damage also spreads to neighboring areas and white matter tracts connected to the lesion. As a consequence of this process, a degeneration of homotopic contralesional sites has been observed [6]. (2) Recent works have evidenced the fact that white matter disconnection can be a good predictor of brain malfunctioning and recovery [54,55,56]. Cheng and colleagues (2020) have demonstrated that degenerative processes in stroke patients occur *progressively* after the stroke, affecting not only the ipsilesional hemisphere but also the contralesional one by means of transcallosal fiber degeneration. The authors observed that the ipsilesional cortex connected to the lesion site showed a significant decrease in cortical thickness compared to the unconnected cortex. Cortical thinning of the ipsilesional cortex connected to the lesion was also accompanied by contralesional homotopic thinning. This degeneration was ascribed to a reduction in the integrity of the transcallosal tracts connecting the homotopic sites [6]. (3) It has been recently shown that the levels of structural integrity in the CC correlate with the motor status of patients depending on the severity of their symptoms. In fact, fractional anisotropy (FA) levels of CC motor section correlate with the motor status of mildly affected patients, while this is not the case in severely affected patients. Conversely, FA levels in the prefrontal section of the callosum correlate with the motor status of severely affected patients, while the same is not true for mildly affected individuals [44,45,46]. These pieces of evidence regarding the role of the CC suggest that such structure needs to be included in the clinical picture when evaluating stroke patients. Furthermore, this evidence also suggests that the CC might have an integrative function in the interhemispheric interplay. In this regard, despite the evidence in support of inhibitory mechanisms supported by the callosum, it has been lately claimed that transcallosal pathways mediate both inhibition and excitation between the hemispheres, and both mechanisms are employed to sculpt hemispheres’ contributions to a given function [39]. In this context, given the evident relevance of different sections of the callosum in the recovery of differently affected patients, we propose that the unaffected hemisphere generally sustains an integrative function in favor of the contralateral one, and this contribution will occur via different transcallosal pathways, depending on spared callosal structures. In this regard, interpreting the inter-hemispheric imbalance in terms of competition between the hemispheres does not hold up: meta-analyses results do not justify the administration of stimulation protocols inspired by the competition model, while improvements after protocols facilitating either the affected hemisphere or the unaffected one have been found [28,47]. Moreover, the competition model would predict that inter-hemispheric imbalance dominates communication after the lesion. However, no evidence of inter-hemispheric imbalance has been found in stroke patients [28]. Furthermore, interpreting the role of the contralesional hemisphere in a competitive manner does not explain how the non-paretic hand in strokes shows slight impairments compared to controls (e.g., [40]). Finally, the negative relationship found between integrity in the CC and motor status of the patients suggests that the better the interhemispheric communication, the better the recovery [39,44,45,46].

This relationship between the integrity in different sections of the CC and the motor status of patients suggests that different compensatory mechanisms take place depending on stroke severity. This distinction between highly and moderately affected patients was first proposed by Di Pino and colleagues (2014). However, the latest negative evidence about the competition model’s assumptions suggests that their model is inaccurate. Further, the bimodal balance–recovery model is currently incomplete because it does not consider (1) the CC in the estimation of structural reserve and its inhibition function, as Carson does; (2) diaschisis processes after stroke, which progressively occur after the stroke and interfere with plastic reorganization; and (3) the role of the prefrontal cortices in network re-organization.

Anticipating the new framework that will be extensively described later in this review, we can anticipate the relevance of the CC in mildly and severely affected patients’ recovery, at this stage of this review, as follows: since motor impairment also depends on the level of structural damage, less affected patients will suffer from less degeneration in the motor system compared to more severely affected patients (damage levels as assessed via DWI techniques and not mere lesion volume assessment: [57]). Therefore, the motor section of CC might be sufficiently intact to support integrative functions between the two motor cortices in the mildly affected compared to the severely affected patients. Differently, in severely affected patients, motor transcallosal fibers are largely damaged, and, since prefrontal areas are generally the most spared ones after a stroke [58], compensatory mechanisms between hemispheres likely occur via prefrontal transcallosal connections. For this group of patients, the genu of the CC indeed positively correlates in terms of structural reserve with the patients’ motor status (see [46]). Liu and colleagues (2015) additionally found in contralesional frontal cortices of stroke patients the following pieces of evidence: (1) increased FA levels in the white matter tracts in the contralesional medial frontal gyrus, and (2) thalamocortical connections including projections to somatosensory, primary motor, and premotor areas were positively correlated with motor recovery [59]. Furthermore, cortical thickness of frontal cortex in the contralesional hemisphere shows an increase already three months after the stroke [60].

While the role of CC is essential for reorganization in mildly and more severely affected patients, it plays a minor role in the recovery of the minimally affected patients—a further, often overlooked category. These patients show minimal impairment, associated with an increased connectivity between the ipsilesional prefrontal area and the sensorimotor cortex compared to controls (see [7]). Therefore, for the minimally affected patients, it will be the prefrontal cortex of the ipsilesional hemisphere that exerts a compensatory role.

As previously mentioned, the prefrontal cortices exert a functional role in strokes’ recovery, regardless of the level of impairment. Mildly affected patients show different patterns of reorganization processes, depending on the degree to which they can resolve the reorganization within the ipsilesional hemisphere. Regardless of their degree of reliance on the ipsilesional or contralesional hemisphere, the importance of the prefrontal cortices’ role in mildly affected patients’ recovery has also been assessed. In fact, in mildly affected patients, it has been found that the stronger the connectivity between prefrontal and motor cortices at rest, the better the motor production (e.g., [8]). Finally, also in severely affected patients, the prefrontal cortices seem to have a role in patients’ recovery. This is not only inferred by the role of the prefrontal CC in the recovery of this group of patients [46]. In fact, severely affected patients also show a correlation between the level of prefrontal–sensorimotor cortices connectivity at rest and their level of motor recovery (e.g., [61]). These different patterns of support and compensation provided by the prefrontal cortices have not been considered thus far, neither in the stratification of stroke patients nor in any of the previous models. The role of prefrontal cortices in stroke recovery has been shown to be critical in all stroke patients. In fact, prefrontal areas seem to exert a different grade of high-level control over motor planning and production depending on the level of impairment of the patient (e.g., [7,8,62,63,64]. In this regard, it is interesting to note that a potential compensatory role of the prefrontal cortices has been observed also in elderly healthy subjects. In fact, an increase in the coupling between prefrontal areas and areas involved in a task predicts their performance in the task [65,66]. In particular, a prefrontal–premotor–M1 coupling predicts psychomotor speed in the elderly during motor execution [67]. The evidence reported in this section is essential to stress the gaps in previous models attempted at explaining stroke interhemispheric communication. In particular, we hope it is now clear to the reader that the roles of the CC and prefrontal cortices cannot be neglected when investigating stroke patients’ recovery. All these pieces of evidence will be integrated and synthetized in a new, more comprehensive framework.

## 5. A New Framework for Predicting Rehabilitation Protocols Effects

Since the first studies on stroke patients, epistemological mistakes have been made in interpreting the relationship between brain re-organization and recovery. J. Williamson recently stressed how clinical medicine is full of examples where causal relationships between phenomena are inferred only on the basis of correlations, while the mechanism behind them cannot be explained, making it difficult to assess the real direction of the relationship [68]. In the case of stroke literature, full recovery has been associated to a return to brain patterns more similar to controls’, while the impossibility to reach full recovery in severe patients has been interpreted as a byproduct of functional imbalance between hemispheres. This interpretation might be the product of a spuriously inferred causal relationship: over-recruitment of the contralesional hemisphere can also be interpreted as a byproduct of a larger damage in severe patients. In these patients, contralesional over-recruitment might be the most efficient strategy to compensate for damage. Di Pino and colleagues (2014) proposed that severely affected patients might benefit from such re-organization [9]. However, their model lacks of an explanation of how brain networks re-adapt according to the level of damage.

**Figure 1:**
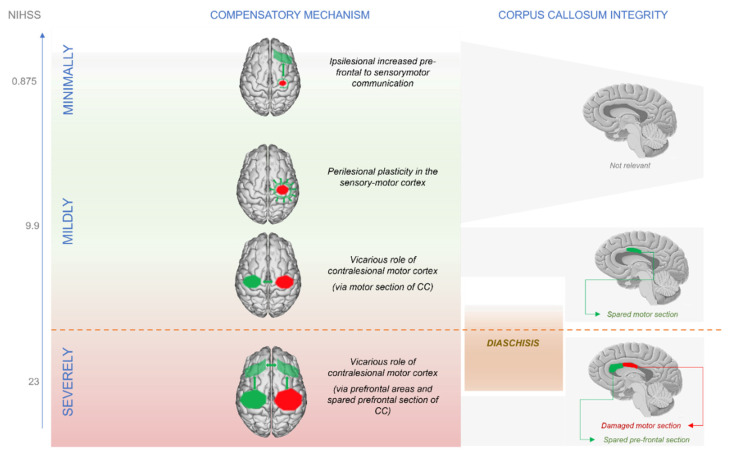
Patients lie on a continuum of severity. Minimally affected patients resolve the competition within the ipsilesional hemisphere. Going towards the more affected patients, the contralesional hemisphere becomes more and more relevant in compensatory processes. The process of diaschisis start playing role in more severe-like mildly affected patients.

In our framework, patients are allocated on a continuum of motor impairment (see Figure 1). Depending on their position on the severity scale, they will probably undergo one re-configuration of brain networks rather than another. Such re-configurations are differing in two functional mechanisms: (1) the role of the prefrontal cortices in exerting high-level motor planning and execution and (2) the relevance that the callosum assumes into sustaining interhemispheric integration.

**Figure 2:**
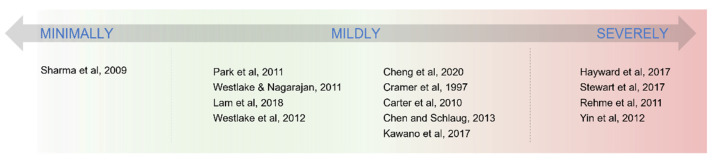
List of references describing patients with different functional and structural connectivity reorganization depending on the level of impairment. Mildly affected patients, in the middle, can either develop in a more “minimally affected” fashion or, on the contrary, can develop towards patterns more similar to severely affected patients.

Minimally affected patients, who show scores very close to controls during the chronic stage, do not rely on the contralesional hemisphere as more affected patients do. In fact, minimally affected patients have sufficient structural reserve in the ipsilesional hemisphere, and this resource allows them to accomplish recovery by relying on increased connectivity between ipsilesional sensorimotor and prefrontal cortex contralateral to the movement, analogously to healthy controls. Contralesional over-recruitment has been proposed to be adaptive in the first days after stroke occurrence [69,70]. After the acute stage, patients with a very good recovery (as the minimally affected ones) return to normal patterns, and the neurophysiological difference remaining between them and age-matched controls is detected in a connectivity increase between prefrontal and sensorimotor areas [7]. For these minimally affected patients, high-level control of motor planning seems sufficient for regaining motor functions (see Figure 1 and Figure 2). Sharma and colleagues show that the increase in connectivity between ipsilesional prefrontal and sensorimotor cortices emerges in the contrast between minimally affected strokes and age-matched controls only in a task of motor imagery, while this difference does not show up in the motor execution condition. In this regard, it should be noticed that healthy controls for stroke patients are always selected on the basis of the “age-matched” criterion. Since stroke patients are usually in their elderly, age-matched controls will show compensatory mechanisms that are typical of older participants. In fact, elderly healthy subjects show an increase in connectivity between prefrontal and sensorimotor cortices, and the level of this increase predicts their psychomotor speed (e.g., [67]). At the same time, healthy participants usually show lower levels of connectivity between prefrontal and sensorimotor cortices in imagery tasks compared to actual motor execution [71,72]. For this reason, the compensatory mechanism in minimally affected strokes emerges only in the imagery condition and not in the executed movement, since their age-matched controls use a similar compensatory strategy that involves prefrontal cortices in the motor execution condition. In this regard, it should be also noticed that becoming an expert through training in a certain motor task is associated with a decrease of cross-regional connectivity in alpha.. In fact, novices need to “train” communication between regions involved in a task before being able to implement a trained behavior in an automatic way [73]. Stroke might be working in the same way: because the compensatory increase in connectivity between ipsilesional prefrontal and sensorimotor areas needs to be trained, the patients show an increase in inter-regional communication compared to controls.

Mildly affected patients are the most difficult to classify in terms of recovery during the acute stage. They lie in the middle section of the continuum (see Figure 1) and thus might develop their recovery course either by getting closer to the “minimally” affected reorganization or towards the “severely affected” one. In the first scenario, some patients might leverage peri-lesional plastic changes and increased connectivity between ipsilesional prefrontal and sensorimotor areas, coming to resemble minimally affected patients. In this case, just as in minimally affected patients, a positive relationship was found between ipsilesional increase in connectivity of the prefrontal cortex and motor status of patients [74]. Plastic processes appear to peak during the acute stage and get arrested at the dawn of the chronic phase [75]. Therefore, especially in the evaluation of mildly affected patients, time elapsed from stroke is crucial. During the chronic phase, mildly affected patients who did not manage to resolve compensation within the ipsilesional hemisphere need to evolve according to a second type of reorganization. This scenario involves a larger relay on the contralesional hemisphere, turning the status of the motor CC into a key factor for patients’ recovery. For these patients, indeed, it might be safely assumed that (1) the extension of the lesion is limited, (2) the degeneration in the motor section of the CC is also consequently lower, and (3) this major preservation of CC might be associated with an analogous preservation of homologous contralesional areas [6]. In this context, the positive correlation between structural reserve in the CC motor section and their motor status provides evidence that interhemispheric integration occurs via the CC motor section. This hypothesis is further corroborated by other contributions investigating functional changes in strokes and showing that mildly affected patients in their chronic phase show a positive relationship between increased interhemispheric sensorimotor communication and motor recovery (e.g., [45,62,76,77,78]. This suggests that these patients manage to cope with the lesion by relying on the contralesional sensorimotor areas that compensate for the ipsilesional damaged ones (see Figure 1). Additionally, high-level control of the motor outcome through prefrontal areas still seems to play a role in mildly affected patients, since increased connectivity at rest within the attention network correlates with their motor recovery [62].

In severely impaired patients, FA in the motor CC does not correlate with motor status, but FA in the prefrontal section of the CC does [46], together with FA in the CST [44]. Therefore, interhemispheric communication does not apparently occur via the motor section of CC but finds rather a less damaged pathway. In this regard, there is evidence that cortical prefrontal areas are more likely preserved from stroke than motor regions [58], and hence the prefrontal section of CC should also be more likely spared since this section of white matter would not connect to any lesioned site. For this reason, the positive correlation between prefrontal CC and motor status in these patients can be interpreted as evidence that the contribution from the spared hemisphere is mediated via the prefrontal tract of the CC, reaching the spared neuronal population of the CST. In fact, the CST is the other structure whose structural integrity correlates with the motor status of highly affected patients. Moreover, there is evidence from “in vivo” studies in monkeys and TMS trials in humans that prefrontal areas communicate with both premotor and motor regions [46,79,80]. From a functional point of view, prefrontal and sensorimotor connectivity of the contralesional hemisphere at rest correlates with motor status in severely affected patients [8]. Finally, Rehme and colleagues (2011) have reported an over-recruitment of the contralesional hemisphere both at the level of the prefrontal areas and sensorimotor areas. This over-recruitment correlates with better motor outcomes in severe patients compared to less impaired ones. This suggests that the more the damage, the more the patient will rely on the contralesional hemisphere, which will communicate with the affected one via prefrontal connections [46] (see Figure 1 and Figure 2). Yin and colleagues have found a significant decrease of connectivity within the sensorimotor network in completely paralyzed patients. Given the scarce interhemispheric sensorimotor communication, it could be argued that these patients cannot even recover a motor plan formulated within the contralesional hemisphere. However, in their partially paralized group, a decrease in connectivity between sensorimotor and prefrontal areas was detected [61]. These results could be interpreted in our new framework as follows: Completely paralyzed patients cannot achieve recovery because of a lack of communication within the sensorimotor network. In fact, their damage might have been so extended to generate diaschisis processes. This phenomenon leads in turn to degeneration of contralesional homotopic areas [6], thus impeding the contralesional hemisphere to communicate motor plans to the ipsilateral prefrontal areas. These regions should be in charge of exploiting the frontal CC to communicate with the ipsilesional hemisphere. Instead, the communication internal to the sensorimotor network seems more spared in the partially paralyzed group, even though a proper high-level control coming from the prefrontal cortices is lacking. For this reason, these patients can achieve some recovery (like in mildly affected strokes) but lack the support of the prefrontal areas.

To summarize (see Figure 1 and Figure 2), we propose that minimally affected patients will rely on a reorganization more similar to healthy controls, where the prefrontal cortex’ increased communication with ipsilesional sensorimotor areas suggests a compensatory role. Mildly affected patients lie in between minimally and severely affected ones, and their reorganization patterns can either tend towards peri-lesional plastic changes in the ipsilesional hemisphere (thus resembling minimally affected patients) or, in case of moderately severe damage, increased interhemispheric connectivity within the sensorimotor network (sustained by the motor CC). Finally, severe patients will rely on the contralesional hemisphere, both at the level of the sensorimotor areas and prefrontal cortex.

## 6. Addressing the Need of New Rehabilitation Protocols

Synchronization of the oscillatory signal between different brain regions has been proposed to support integration of information (e.g., [81,82,83]). In recent years, a new technique aiming at increasing oscillatory cortico-cortical coherence has been found in the so-called paired associative rTMS. In this regard, already in 2008, Plewnia and colleagues showed synchronous bifocal rTMS as an effective tool to increase long-range cortico-cortical coherence. They found that application of simultaneous high frequency (10 Hz) rTMS over both the motor and occipital cortex increases their spatio-temporal coherence in alpha and lower beta band in the stimulated hemisphere [84].

**Figure 3:**
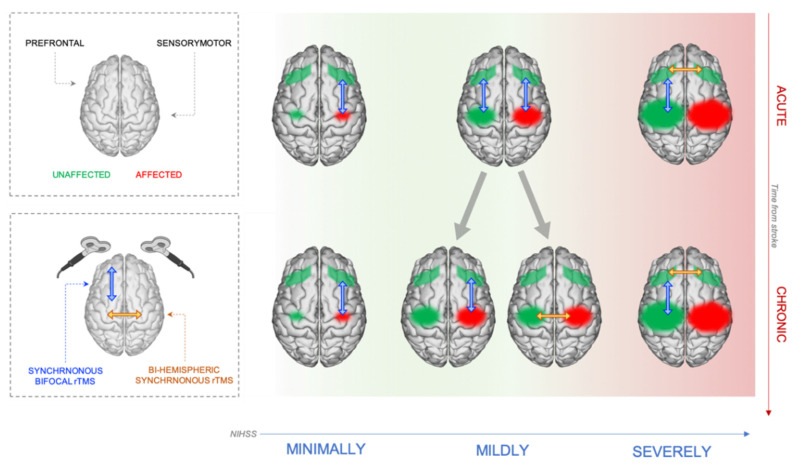
Scheme of the NIBS stimulation protocols developed on the basis of the patient’s individual brain reorganization proposed in the framework.

According to our framework, we propose that in minimally affected patients, bifocal rTMS can be used to increase coherence between sensorimotor and prefrontal areas within the ipsilesional hemisphere (see Figure 3). This protocol could be administered already from the acute phase. A patient who scores as minimally affected on the assessment scales already during the acute phase will unlikely get worse over time and will thus present a re-organization where prefrontal high-level motor control supports further motor recovery.

Differently from the minimally affected patients, distinct protocols should be administered to mildly affected patients, depending on the time passed from the stroke (see Figure 3). During the acute phase, the kind of re-adaptation process the patient will develop towards the chronic phase cannot be reliably predicted. At this stage, the only certainty is the supportive function of prefrontal areas after stroke. Thus, the safest procedure is a communication reinforcement between prefrontal areas and the sensorimotor areas. To this aim, bifocal rTMS can be used to increase synchronicity in alpha and low beta frequency bands, targeting the prefrontal and sensorimotor areas of both hemispheres. Then, once the patient has reached the chronic stage, EEG measures can help to differentiate between patients who managed to recover by exploiting peri-lesional plasticity and high-level support from frontal areas and patients who rely more on contralesional sensorimotor regions. In this regard, it should be found that the first type of patients shows an increased coherence between ipsilesional prefrontal and sensorimotor areas compared to healthy controls, while the second type shows increased interhemispheric coherence within the sensorimotor network. In the first case, we would be inclined to boost the afore mentioned synchronicity and help the patient to reinforce the increased coherence between prefrontal and sensorimotor areas of the damaged hemisphere. In the second scenario, we would be inclined to exploit bihemispheric synchronous rTMS to increase interhemispheric synchronicity within the sensorimotor network (see [85]).

We are aware that, on one hand, rehabilitation procedures requiring the comparison between the patient status and an age-matched healthy control might not be always feasible in the clinical practice. On the other hand, our framework provides the possibility to optimize the patient rehabilitation protocol by exploiting specific electrophysiological features. Given the potential return in term of recovery efficiency, we believe that an open, general, and normative database of EEG recordings, possibly already equipped with standardized coherence analysis pipelines, would be highly beneficial for predicting stroke development.

Finally, severely affected patients should be trained to recruit vicarious areas from the contralesional hemisphere during a motor task (see Figure 3). Already during the acute stage, patients with great damage and functional impairment should be reinforced into recruiting prefrontal areas, since they would benefit from a greater interhemispheric coherence at the level of prefrontal cortices. In fact, even at anatomical level, this group shows a positive relationship between the levels of structural integrity in the prefrontal callosum and motor recovery [46]. Improving this communication means that high-level areas in the contralesional hemisphere can send motor programs to the ipsilesional prefrontal cortex. However, during the acute stage a patient showing a great level of damage might go through re-adaptation processes that are more similar to those of mildly affected patients coping with lesion damage through an increased interhemisperical sensorimotor communication. For this reason, we propose that severe patients who show reorganization patterns more similar to the ones of mildly affected patients should benefit from protocols aimed at boosting high-level motor control. On the other hand, in case a severe patient shows an outcome close to the end of the motor score continuum, clinicians should boost both interhemispheric prefrontal communication and connectivity between contralesional prefrontal and sensorimotor cortices already during the acute stage. This would be performed via bihemispheric synchronous stimulation on the prefrontal cortices and bifocal intrahemispheric synchronous stimulation over the prefrontal and sensorimotor cortices of contralesional hemisphere, respectively. In this case as well, the bifocal stimulation could be a starting point to develop a protocol useful to boost synchronization in severely affected strokes [84].

In delineating this individualized time-dependent stimulation rehabilitation protocols, we followed the Stroke Roundtable Consortium guidelines on post-stroke phases [86]. Moreover, as we are aware that a single stimulation session would not be enough in order for the effectiveness of the protocol to be assessed [87], we suggest the administration of at least five consecutive sessions before re-assessing motor impairment levels.

Finally, it is worth it to stress again that, in order to accomplish such an individualized stimulation treatment on stroke patients, EEG connectivity measures would be needed in order to be sure about the undertaken re-organization. While assessment scores can give good insights on this, given previous literature assessing a relationship between impairment levels and connectivity patterns, it would be safer to compare individual connectivity patterns with the ones of controls. Therefore, providing clinicians with a fast methodology that allows for comparison of the patient’s EEG patterns with normal connectivity ones is a crucial to-be-addressed issue in clinical neuroscience research.

## 7. Conclusions

In this work, a new framework of stratification for stroke patients was proposed, aided to a better patient-dependent choice of rehabilitation stimulation protocols. After discussing the limits of models currently available for choosing rehabilitation strategy, we showed how some relevant structural and functional aspects have been disregarded thus far. For this reason, we propose a new, more comprehensive classification of stroke patients, based on both impairment severity and structural/functional outcomes. Within this new framework, we outline a possible strategy for patient-dependent rehabilitation stimulation protocol development. Stroke patients should be considered over a continuum of impairment severity. Depending on impairment level, the patient will undergo different compensatory routes. These options differ depending on the role of the callosum into sustaining interhemispheric integration and on the role of prefrontal cortices high level in controlling motor planning and production. The convention that well-recovered patients resemble healthy controls in brain reorganization and consequently all patients should be pushed towards the same, healthy-like state does not hold up. Patients should be supported in their individual re-organization, which critically depends on the available structural resources. We believe that our framework sheds new light into recovery mechanisms after a stroke and provides a novel approach to improve individualized, patient-dependent rehabilitation protocols.
